# Silica-gel Particles Loaded with an Ionic Liquid for Separation of Zr(IV) Prior to Its Determination by ICP-OES

**DOI:** 10.3390/s16071001

**Published:** 2016-06-29

**Authors:** Hadi M. Marwani, Amjad E. Alsafrani, Abdullah M. Asiri, Mohammed M. Rahman

**Affiliations:** 1 Chemistry department, Faculty of Science, King Abdulaziz University, P.O. Box 80203, Jeddah 21589, Saudi Arabia; mjad.1985@hotmail.com (A.E.A.); ceamr2@gmail.com (A.M.A.); 2 Center of Excellence for Advanced Materials Research (CEAMR), King Abdulaziz University, P.O. Box 80203, Jeddah 21589, Saudi Arabia

**Keywords:** solid-phase extraction, ionic-liquid, Zr(IV) detection, adsorption isotherm, inductively coupled plasma-optical emission spectrometry (ICP-OES)

## Abstract

A new ionic liquid loaded silica gel amine (SG-APTMS-N,N-EPANTf_2_) was developed, as an adsorptive material, for selective adsorption and determination of zirconium, Zr(IV), without the need for a chelating intermediate. Based on a selectivity study, the SG-APTMS-N,N-EPANTf_2_ phase showed a perfect selectivity towards Zr(IV) at pH 4 as compared to other metallic ions, including gold [Au(III)], copper [Cu(II)], cobalt [Co(II)], chromium [Cr(III)], lead [Pb(II)], selenium [Se(IV)] and mercury [Hg(II)] ions. The influence of pH, Zr(IV) concentration, contact time and interfering ions on SG-APTMS-N,N-EPANTf_2_ uptake for Zr(IV) was evaluated. The presence of incorporated donor atoms in newly synthesized SG-APTMS-N,N-EPANTf_2_ phase played a significant role in enhancing its uptake capacity of Zr(IV) by 78.64% in contrast to silica gel (activated). The equilibrium and kinetic information of Zr(IV) adsorption onto SG-APTMS-N,N-EPANTf_2_ were best expressed by Langmuir and pseudo second-order kinetic models, respectively. General co-existing cations did not interfere with the extraction and detection of Zr(IV). Finally, the analytical efficiency of the newly developed method was also confirmed by implementing it for the determination of Zr(IV) in several water samples.

## 1. Introduction

Room temperature ionic liquids (RTILs) have been considerably used and there is growing interest in them in different chemistry fields. Organic, inorganic, analytical and electrochemistry are common areas for application of ionic liquids (ILs) [1,2]. In addition, ILs have received significant attention in analytical chemistry as environmentally-friendly solvents, replacing volatile, toxic hazardous and flammable organic compounds [3]. Thus, they have been used in solvent extractions [4], solid phase extraction [5,6,7], gas chromatography [8], liquid chromatography [9], capillary electrophoresis [10], mass spectrometry [11], electrochemistry [12], sensors [13] and spectroscopy [14]. Several research studies have been consecrated to examining and studying the spectroscopic and physico-chemical properties of ILs and designing of new families of chiral and achiral ILs [15,16,17,18,19]. Furthermore, ILs have many fascinating properties resulted in a wide range of promising applications in which they have been employed in various fields [20,21].

Extraction systems for metal ions based on ILs by various extraction techniques are a very active field in analytical chemistry [22,23,24,25]. Extraction of metal ions by various extraction techniques such as liquid-liquid extraction (LLE) [26] and solid phase extraction (SPE) [27,28], is a necessary step prior to their detection by a suitable analytical technique. Direct detection of metal ions by analytical techniques such as flame-atomic-absorption spectrometry [29] and ICP-OES [5] has some drawbacks due to the high levels of chemical interferences that usually accompany the analyte, thus leading to a lack of instrumental sensitivity [30].

For the separation of metal ions from various matrices using ILs (as extraction solvents), ILs depend essentially on the incorporation of chelating agents to form metal complexes in order to increase the hydrophobicity in the ions, and the last step (transfer and partition) represents the formation of a metal complex between the two phases [6]. According to the literature, a liquid-liquid extraction system was expanded for the isolation of Ni(II), Cu(II) and Pb(II) from water using a newly synthesized ionic liquid, 1-butyl-3-methylimidazolium hexafluorophosphate ([BMIM][PF_6_]), combined with the ligand 2-aminothiophenol [31]. A straightforward and efficient technique was proposed for the extractive preconcentration of U(VI) in seawater using the ionic liquid 1-octyl-3-methylimidazolium hexafluorophosphate ([C_8_mim][PF_6_]) as a unique phase for liquid–liquid extraction of U(VI) combined with dimethylphenylazosalicylfluorone as a new reagent [32].

The main obstacle for using imidazolium ionic liquids, i.e., 1-alkyl-3-methyl-imidazolium hexafluorophosphate ([C_n_mim][PF_6_]) or 1-alkyl-3-methyl-imidazolium bis(trifluoromethane- sulfonyl)imide ([C_n_mim][Tf_2_N]) in IL-based extraction systems is the loss of their selective cations/anions into the active phase through extraction [22,33,34]. As a result, the ILs will decomposed and pollut the aqueous phase [34,35]. Moreover, a reported study has described that some imidazolium based ILs showed higher toxicity as compared to common organic solvents on bacteria in industrial wastewater treatment systems [36]. As it is well known, the properties/behaviors of ILs are easily regulated by joining variant active cations/anions which can overcome the disadvantages of IL-based extraction [34].

The shortcomings in applications of ILs for metal ions extraction are in their higher viscosity that causes a low mass-transfer rate, long time for reach equilibrium, complexity of the isolate phase and loss of ILs into the active aqueous phase [37]. These limitations can be overcome by immobilizing the ionic liquid on a solid support (known as supported ionic liquid phase, SILP). Different types of SILP materials such as polymers [38] and silica [39] have been used in SPE. However, silica gel (SG) has emerged as the most appropriate solid support due to its large specific surface area and high porosity [40,41]. Furthermore, the modified SG has a good thermal stability as well as high effectiveness for selective separation of metal ions from different matrices [7].

In accordance, the present study was devoted to develop a new SILP via a hybrid combination of SG chemically bound amine, 3-aminopropyltrimethoxysilane (APTMS), as a silane coupling agent, with the new ionic liquid (N,N-EPANTf_2_). The aim of this study was also focused on the utilization of N,N-EPANTf_2_ as an extraction medium for metal ions. The major advantage of the newly modified SG-APTMS-N,N-EPANTf_2_ for extraction of Zr(IV) was the presence of amino sites along with donor atoms present in N,N-EPANTf_2_, according to Pearson’s hard and soft acid-base theory [42]. Several parameters controlling the maximum uptake of Zr(IV) by SG-APTMS-N,N-EPANTf_2_ were investigated in a batch system mode. Adsorption isotherms and kinetic data were evaluated by well known models. In order to assert the applicability of the suggested technique to real samples, the effect of medium compositions containing various interfering ions was also studied. Finally, the extended technique was applied to the selective detection and quantification of Zr(IV) ions in various real water samples.

## 2. Materials and Methods

### 2.1. Chemicals and Reagents

Bis(trifluoromethane)sulfonimide lithium (LiNTf_2_), N,N-diethyl-*p*-phenylenediamine monohydro- chloride (N,N-EPACl), toluene, hydrochloric acid, ethyl alcohol (EtOH) and diethyl ether were purchased from Sigma-Aldrich (Milwaukee, WI, USA). SG (SiO_2_, micorparticle size 10–20 nm, purity of 99.5%), 3-aminopropyltrimethoxysilane (APTMS), mercuric nitrate [Hg(NO_3_)_2_], sodium selenite [Na_2_SeO_3_] as well as 1000 mg·L^−1^ Au(III), Cu(II), Co(II), Zr(IV), Cr(III) and Pb(II) stock standard solutions were also obtained from Sigma-Aldrich. 

### 2.2. Preparation of the New Adsorbent Based on Silica Gel

#### 2.2.1. Preparation of N,N-EPANTf_2_ Ionic Iiquid

The synthesis of N,N-EPANTf_2_ ionic liquid was previously reported by Marwani [15,43]. Briefly, an aqueous solution of N,N-EPACl (1.00 g) was mixed with a solution of LiNTf_2_ (1.43 g) in doubly distilled deionized water. The mixture was stirred for 2 h at room temperature, and the reaction produced two layers. Finally, separation of the two layers was carefully achieved, and the lower layer was dried under vacuum overnight. 

#### 2.2.2. Activation of SG

SG powder (25.0 g) was suspended in 50% (v/v) HCl solution (200 mL), and the mixture was refluxed under stirring for 8 h, to avoid the existence of nitrogenous impurities or metal oxides as well as enhancing silanol group content on the SG surface. The activated SG powder was filtered and washed several times with doubly distilled deionized water until neutralization. Finally, it was dried in an oven at 120 °C for 5 h for removing any adsorbed water on the SG surface. 

#### 2.2.3. Synthesis of SG-APTMS

For preparation of SG chemically bound amine, activated SG (8 g) was suspended in toluene (100 mL) in a round bottom flask, and then 3-aminopropyltrimethoxysilane (APTMS, 6 mL) was gradually added to this suspension as a silane coupling agent. The reaction mixture was allowed to reflux with continuous stirring for 12 h at ~100 °C. The final product was filtered and washed with toluene, ethanol and diethyl ether, then, it was dried in an oven at 80 °C for 8 h to obtain SG chemically bound amine (SG-APTMS). 

#### 2.2.4. Synthesis of SG-APTMS-N,N-EPANTf_2_

**Scheme 1:**
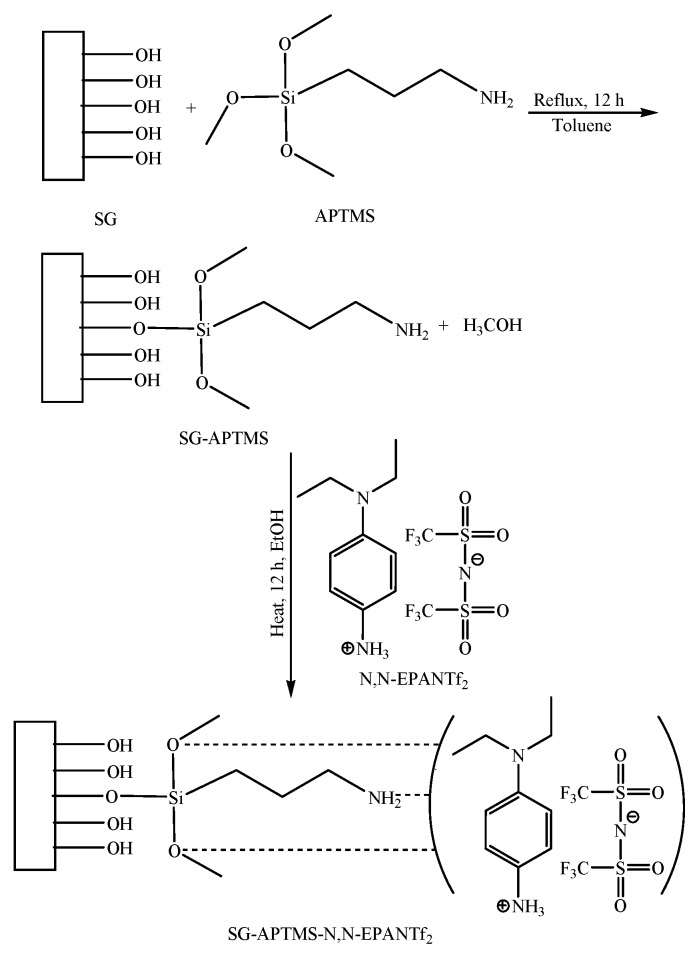
Synthetic route of SG-APTMS-N,N-EPANTf_2_ phase.

SG-APTMS-N,N-EPANTf_2_ was prepared by functionalizing the hydrophobic ionic liquid (N,N-EPANTf_2_) on the surface of SG-APTMS. An amount of N,N-EPANTf_2_ (1.5 g) was completely dissolved by warming in ethanol (50 mL) and added to a suspension of SG-APTMS (5 g) in ethanol (60 mL). The reaction mixture was then stirred for 12 h at 60 °C. The modified phase (SG-APTMS-N,N-EPANTf_2_) was filtered and washed with ethanol, doubly distilled deionized water and diethyl ether. The SG-APTMS-N,N-EPANTf_2_ phase was then dried in an oven at 60 °C for 8 h and kept in a desiccator. Scheme 1 illustrates the synthetic route used to prepare the SG-APTMS-N,N-EPANTf_2_ adsorbent.

### 2.3. Batch Procedure

In this investigation, the effect of various parameters including pH, initial concentration of Zr(IV) and contact time on the adsorption of SG-APTMS-N,N-EPANTf_2_ towards Zr(IV) were studied For the effect of pH on the SG-APTMS-N,N-EPANTf_2_ selectivity towards different metal ions, 2 mg·L^−1^ standard solutions were prepared with pH values ranging from 1.0 to 9.0 with a series of appropriate buffer solutions, (0.2 mol·L^−1^ HCl/KCl) for pH 1.0 and 2.0, (0.1 mol·L^−1^ CH_3_COOH/CH_3_COONa) for pH 3.0–6.0 and (0.1 mol·L^−1^ Na_2_HPO_4_/HCl) for pH 7.0–9.0. Then, an amount of SG-APTMS-N,N-EPANTf_2_ (20 mg) was individually mixed with each standard solution and mechanically shaken for 1 h by use of a mechanical shaker at 150 rpm and 25 °C.

The supernatant concentrations of residual metal ions were determined by ICP-OES after filtration. For estimating the uptake capacity of SG-APTMS-N,N-EPANTf_2_ towards Zr(IV), selected concentrations (0, 5, 10, 20, 30, 40, 60, 80, 100, 125, 150 and 200 mg·L^−1^) of Zr(IV) were prepared at the optimum pH value of 4.0 and individually mixed with SG-APTMS-N,N-EPANTf_2_ (20 mg). The mixtures were mechanically shaken for 1 h at 25 °C. In addition, the effect of contact time on the SG-APTMS-N,N-EPANTf_2_ adsorption capacity for Zr(IV) was investigated as above at equilibrium periods of 2.5, 5, 10, 20, 30, 40, 50, 60, 75 and 90 min.

### 2.4. Instrumentation

A Perkin Elmer Spectrum 100 series FT-IR spectrometer (Beaconsfield, Bucks, UK), SEM on a field emission-scanning electron microscope (FE-SEM, QUANT FEG 450, Amsterdam, The Netherlands) and a Barnstead Thermolyne 47900 benchtop muffle furnace (Waltham, MA, USA) were used to characterize the newly synthesized phase. A Jenway model 3505 laboratory pH meter (CamLab, Cambridge, UK) was used for the pH measurements. In addition, a Perkin Elmer ICP-OES model Optima 4100 DV (Norwalk, CT, USA) was used to perform measurements of metal ions concentration at the selected wavelengths of 267.50, 327.39, 238.89, 343.82, 267.72, 220.35, 196.026 and 194.17 nm for Au(III), Cu(II), Co(II), Zr(IV), Cr(III), Pb(II), Se(IV) and Hg(II), respectively. All instrumental parameters used in this study were similar to those applied in our previous study [44].

## 3. Results and Discussion

### 3.1. Characterization of the SG-APTMS-N,N-EPANTf_2_

#### 3.1.1. Surface Coverage Value of the SG-APTMS-N,N-EPANTf_2_ Phase

SG-APTMS-N,N-EPANTf_2_ (100 mg) was placed in a dry porcelain crucible, gradually heated into a furnace from 50 to 700 °C and kept at this temperature for 1 h. The remaining SG-APTMS-N,N-EPANTf_2_ phase was left to cool in a desiccator and weighed to determine the mass of desorbed N,N-EPANTf_2_. The hydrophobic ionic liquid weight loss was determined by variation of sample masses. The concentration of N,N-EPANTf_2_ on SG-APTMS surface was found to be 0.46 mmol·g^−1^, based on this thermal desorption method.

#### 3.1.2. FT-IR Analysis

**Figure 1:**
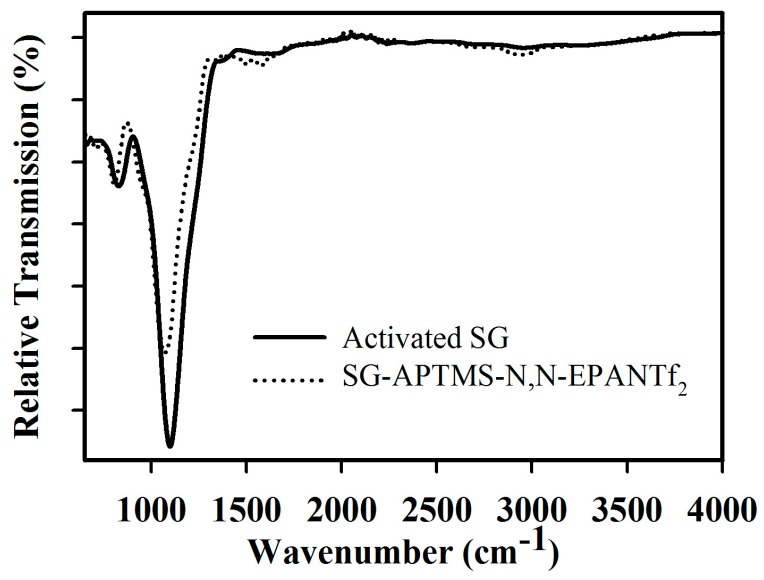
FT-IR spectra of activated SG and SG-APTMS-N,N-EPANTf_2_ phases.

The structure of activated SG and SG-APTMS-N,N-EPANTf_2_ was studied and evaluated by the use of FT-IR spectroscopy. The FT-IR spectrum of newly modified adsorbent exhibited characteristic peaks, related to both APTMS and the N,N-EPANTf_2_ ionic liquid. From Figure 1, it can be observed that several bands appeared at positions of about 735 cm^−1^ for (C-H), 1497 cm^−1^ for (C-S), 1577 cm^−1^ for (C=C) bonds. In addition, a characteristic stretching vibration band for (NH) bonds appeared at 2950 cm^−1^, confirming the presence of the anchored propyl group on SG-APTMS-N,N-EPANTf_2_ adsorbent [43]. 

#### 3.1.3. SEM Analysis

**Figure 2:**
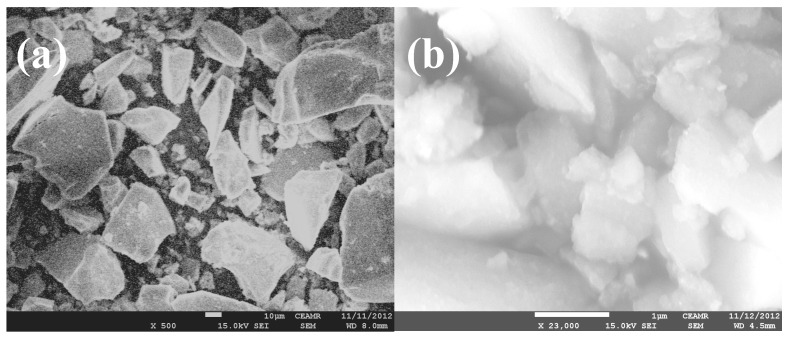
SEM images of (**a**) activated SG and (**b**) SG-APTMS-N,N-EPANTf_2_ phases.

In order to obtain more information about the changes in the surface characteristics of activated SG and SG-APTMS-N,N-EPANTf_2_, SEM images were obtained as presented in Figure 2a,b. As displayed in Figure 2b, N,N-EPANTf_2_ particles that covered the surface of SG chemically bound amine (SG-APTMS) were clearly observed. In addition, SG-APTMS particles were completely coated by N,N-EPANTf_2_. This can be regarded as evidence that the hydrophobic ionic liquid N,N-EPANTf_2_ was successfully loaded on SG-APTMS surface. 

### 3.2. Batch Method

#### 3.2.1. Effect of pH and Selectivity Study

**Figure 3:**
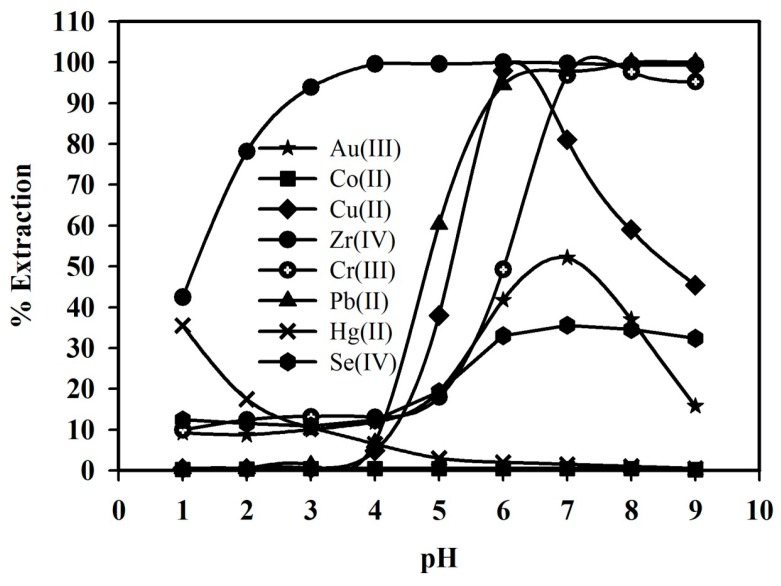
Effect of pH on the adsorption of 2 mg·L^−1^ Zr(IV), Au(III), Cu(II), Co(II), Cr(III), Pb(II), Se(IV) and Hg(II) on 20 mg SG-APTMS-N,N-EPANTf_2_ at 25 °C.

Metal adsorption processes can be influenced by either protonation of binding sites of the adsorbent in an acidic solution or precipitation of many metal ions by hydroxide ions in a basic solution. Therefore, the effect of the pH of aqueous solutions was the first parameter to be evaluated. For investigation of the pH effect on the selectivity of SG-APTMS-N,N-EPANTf_2_ towards various metal ions, including Au(III), Cu(II), Co(II), Zr(IV), Cr(III), Pb(II), Se(IV) and Hg(II), 25 mL of 2 mg·L^−1^ of each selected metal ion sample solution was individually mixed with 20.0 mg of SG-APTMS-N,N-EPANTf_2_ at different pH values (1.0–9.0). As can be noticed from Figure 3, there was no remarkable change in the % extraction of Co(II) and Hg(II). In addition, an increase followed by a subsequent decrease is observed in the % extraction of Au(III), Cu(II), Cr(III), Pb(II) and Se(IV) with an increase of the pH. However, it was interesting to note that the % extraction of Zr(IV) dramatically increased with an increase of the pH value. In sense, the Zr(IV) was found to give the highest quantitative % extraction within the pH range 4.0–9.0. In addition, it can be clearly observed that SG-APTMS-N,N-EPANTf_2_ is most selective towards Zr(IV) at pH 4.0. Therefore, pH 4.0 was selected for the extraction of Zr(IV) as an optimum pH value for further studies of parameters affecting the maximum capacity of SG-APTMS-N,N-EPANTf_2_ for Zr(IV). The highest % extraction of Zr(IV) at pH 4.0 can be ascribed to an excellent affinity of incorporated donor sites (N, O and S) in the chemically bound amine (SG-APTMS) and ionic liquid (N,N-EPANTf_2_), presented in the newly modified SG-APTMS-N,N-EPANTf_2_. 

In addition, results obtained from calculated distribution coefficient (*K_d_*) for each metal ion, as illustrated in Table 1, strongly supported that SG-APTMS-N,N-EPANTf_2_ phase was the highest in selectivity for Zr(IV) over other metal ions included in this study. The distribution coefficient (*K_d_*) can be expressed as follows [45]:

$$K_{d}=\frac{\left(C_{i}-C_{e}\right)}{C_{e}}\times \frac{V}{m}$$

where *C_i_* and *C_e_* represent the initial and final concentrations (mg·L^−1^), respectively, *V* refers to the volume of solution (mL), and *m* indicates the mass of adsorbent (g). From Table 1, it can be clearly noted that the highest *K_d_* value was acquired for Zr(IV), 289.45 × 10^3^ mL·g^−1^, as a representative value for emphasizing the highest incorporated selectivity of SG-APTMS-N,N-EPANTf_2_ phase towards Zr(IV).

#### 3.2.2. Determination of Zr(IV) Adsorption Capacity

**Figure 4:**
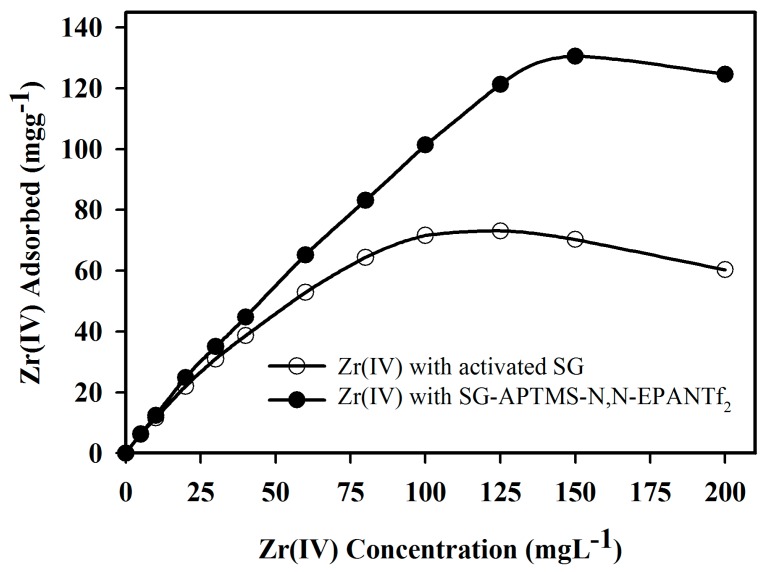
Adsorption profile of Zr(IV) on 20 mg activated SG and SG-APTMS-N,N-EPANTf_2_ in relation to the concentration at pH 4.0 and 25 °C.

To estimate the Zr(IV) adsorption capacity on SG-APTMS-N,N-EPANTf_2_, varied concentrations (0–200 mg·L^−1^) of Zr(IV) in 25 mL aqueous solutions were adjusted to pH 4.0 and individually mixed with 20 mg SG-APTMS-N,N-EPANTf_2_. Figure 4 displays the uptake capacity of SG-APTMS-N,N-EPANTf_2_ towards Zr(IV) obtained from the adsorption isotherm experiment. As can be deduced from Figure 4, the adsorption capacity of SG-APTMS-N,N-EPANTf_2_ was determined to be 130.53 mg of zirconium per gram of adsorbent. This value was also compared with those previously reported for the adsorption capacity of Zr(IV) in other studies (1.15 [27], 50 [46], 86 [47] and 179 [48] mg·g^−1^). Moreover, the adsorption capacity of Zr(IV) on activated SG was calculated to be 73.07 mg·g^−1^ (Figure 4), providing that the uptake capacity for Zr(IV) was improved by 78.64% with newly modified SG-APTMS-N,N-EPANTf_2_ adsorbent.

### 3.3. Adsorption Isotherm Models

**Figure 5:**
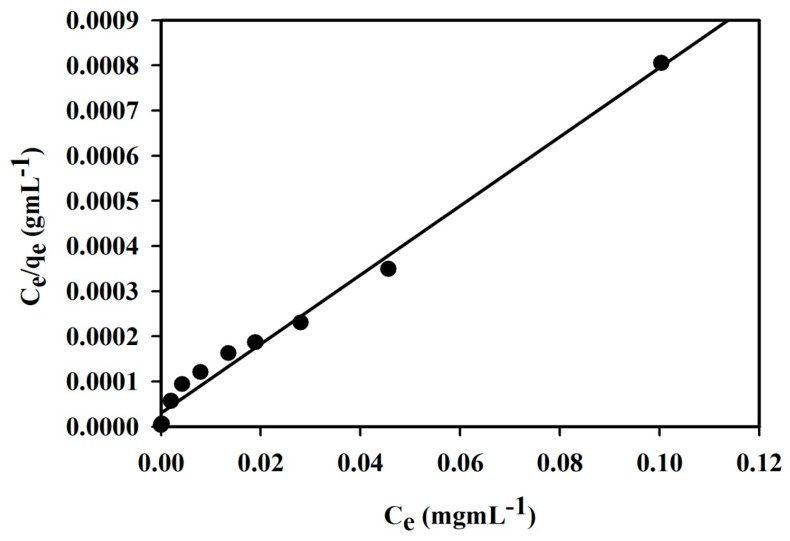
Langmuir adsorption isotherm model of Zr(IV) adsorption on 20 mg SG-APTMS-N,N-EPANTf_2_ at pH 4.0 and 25 °C. Adsorption experiments were performed using different concentrations (0–200 mg·L^−1^) of Zr(IV) under batch conditions.

Adsorption isotherm studies were mainly applied in order to obtain an equation that accurately explained the results. Based on that, well known models describing the adsorption nature on adsorbent were evaluated. The Langmuir adsorption isotherm represents a monolayer adsorption on active sites of the adsorbent. The Langmuir adsorption isotherm equation can be obtained as follows [49]:

*C_e_/q_e_* = (*C_e_/Q_o_*) + 1/*Q_o_b*

where, *C_e_* is the concentration of Zr(IV) in solution at equilibrium (mg·mL^−1^), and *q_e_* refers to the amount of Zr(IV) per gram of SG-APTMS-N,N-EPANTf_2_ adsorbent at equilibrium (mg·g^−1^). Langmuir constants *Q_o_* and *b* correspond to related to the maximum Zr(IV) adsorption capacity (mg·g^−1^) and affinity parameter (L·mg^−1^), respectively. These constants can be estimated from a linear plot of *C_e_/q_e_* versus *C_e_*. Moreover, a dimensionless constant separation factor, *R_L_*, represents intrinsic characteristics of the Langmuir isotherm and can be obtained as follows:

*R_L_* = 1/(1 + *bC*_o_)

where *b* denotes the Langmuir constant and indicates the adsorption nature and the isotherm shape, and *C*_o_ refers to the initial concentration of Zr(IV). The value of *R_L_* describes the nature of the adsorption isotherm, and *R_L_* values usually range between 0 and 1 provide that the adsorption process is favorable [50]. The experimental data of Zr(IV) fitted well the Langmuir equation based on the least squares fit with a correlation coefficient (*R^2^*) value of 0.988 shown in Figure 5, confirming the validity of the Langmuir model. Thus, Langmuir isotherm model indicated that the adsorption process was mainly a monolayer of Zr(IV) on SG-APTMS-N,N-EPANTf_2_ phase. Langmuir constants *Q*_o_ and *b* were determined to be 130.69 mg·g^−1^ and 0.25 L·mg^−1^, respectively. The *R_L_* value of Zr(IV) adsorption on SG-APTMS-N,N-EPANTf_2_ was calculated to be 0.03, providing a highly favorable adsorption process. Moreover, the Zr(IV) adsorption capacity value (130.69 mg·g^−1^) estimated from the Langmuir equation was strongly consistent with that (130.53 mg·g^−1^) determined from the experiment.

### 3.4. Effect of Contact Time

**Figure 6:**
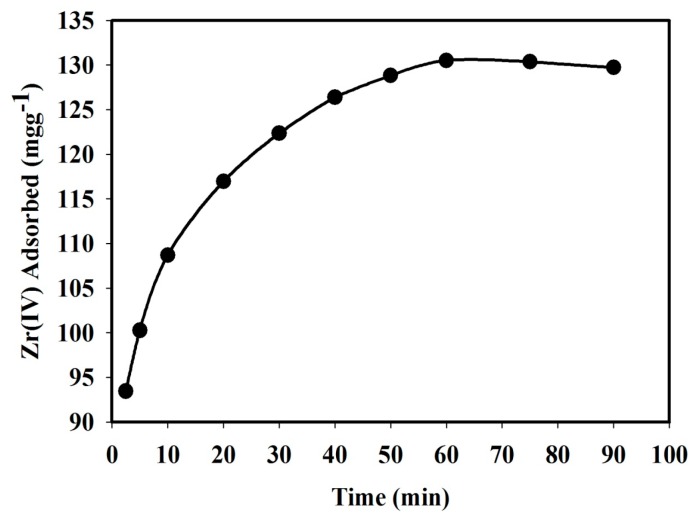
Effect of contact time on 150 mg·L^−1^ Zr(IV) adsorption by 20 mg SG-APTMS-N,N-EPANTf_2_ at pH 4.0 and 25 °C.

The shaking time is an essential factor for estimating the time required for the adsorption process to attain equilibrium. In order to determine the equilibrium time of Zr(IV) on SG-APTMS-N,N-EPANTf_2_, a batch procedure was carried out at different contact times (2.5, 10, 20, 30, 40, 50, 60, 75 and 90 min). Figure 6 presents the contact time profile vs. milligrams of Zr(IV) adsorbed per gram SG-APTMS-N,N-EPANTf_2_. As shown in Figure 6, the adsorption of Zr(IV) increased when the shaking time increased, and equilibrium is attained after 60 min. As can be clearly observed from Figure 6, an amount of 122.36 mg·g^−1^ Zr(IV) was adsorbed on SG-APTMS-N,N-EPANTf_2_ after only 30 min of equilibrium period. The amount of Zr(IV) adsorbed onto SG-APTMS-N,N-EPANTf_2_ phase was also increased until the maximum adsorption capacity (130.53 mg·g^−1^) for Zr(IV) was reached. After this period of time, the adsorbed amount of Zr(IV) on SG-APTMS-N,N-EPANTf_2_ became almost constant. Thus, the results clearly show that the equilibrium kinetics for Zr(IV) adsorption on SG-APTMS-N,N-EPANTf_2_ phase were fast.

#### Kinetic Models

The study of the kinetic mechanism that controls the adsorption process is important for assessment of the time course for the adsorption process. Therefore, experimental data of Zr(IV) adsorption on SG-APTMS-N,N-EPANTf_2_ were analyzed in terms of both pseudo first- and second-order kinetic equations. The pseudo first-order equation can be represented by the following equation [51]:

*log*(*q_e_* – *q_t_*) = logq_e_ – (*k*_1_/2.303)*t*

where, *q_e_* (mg·g^−1^) and *q_t_* (mg·g^−1^) correspond to the amount of Zr(IV) adsorbed at equilibrium and at time (min), respectively. The adsorption rate constant *k_1_* (min^−1^) and adsorption capacity *q_e_* can be calculated from the linear plot of *log(q_e_ – q_t_)* versus *t*. The pseudo second-order equation can be displayed as follows [51]:

$$t/q_{t}=1/\nu_{\circ }+(1/q_{e})t$$

where, $\nu_{\circ }=k_{2}q_{e}^{2}$ is the initial adsorption rate (mg·g^−1^·min^−1^) and *k*_2_ (g·mg^−1^·min^–1^) refers to the rate constant, *q_e_* (mg·g^−1^) corresponds to the amount of Zr(IV) adsorbed at equilibrium, and *q_t_* (mg·g^−1^) denotes the amount of Zr(IV) on the SG-APTMS-N,N-EPANTf_2_ surface at any time (min). From the linear plot of *t/q_t_* versus *t*, kinetic parameters of *q_e_* and $\nu_{\circ }$ can be estimated.

**Figure 7:**
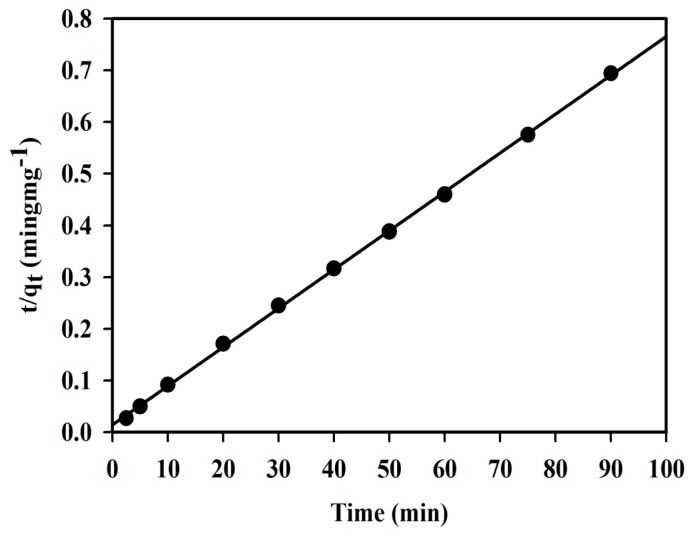
Pseudo second-order adsorption kinetic model of Zr(IV) uptake on 20 mg SG-APTMS-N,N-EPANTf_2_ at pH 4.0 and 25 °C.

From the results, the pseudo second-order equation was found to better represent the adsorption kinetics data than the pseudo first-order equation. From the pseudo second-order equation, the correlation coefficient factor of (*R^2^*) = 0.999 (Figure 7). In addition, parameters *q_e_*, $\nu_{\circ }$ and *k*_2_ were determined to be 133.1 mg·g^−1^, 69.28 mg·g^−1^·min^−1^ and 0.004 g·mg^−1^·min^−1^, respectively. It can be clearly noticed that the value of *q_e_* is relatively close to those acquired from the adsorption isotherm experiments (130.53 mg·g^−1^) and from the Langmuir model (130.69 mg·g^−1^), strongly supporting the validity of the Langmuir isotherm model. 

### 3.5. Performance of the Method in Analytical Applications

#### 3.5.1. Effect of Interfering Ions

The influence of complex matrices on the extraction of Zr(IV) was investigated in order to assess the applicability of the proposed methodology for analytical applications in real samples. For this purpose, 1 mg·L^−1^ Zr(IV) in 25 mL sample solutions were prepared with either individual or mixed interfering ions. According to Table 2, it is motivating to note that Zr(IV) extraction was not influenced by the medium composition despite the presence of major cations like Na^+^, K^+^, NH_4_^+^, Ca^2+^ and Mg^2+^ in 3000-fold excess, Cd^2+^, Cu^2+^ and Pb^2+^ in 500-fold excess, Zn^2+^ in 600-fold excess, Fe^3+^ in 300-fold excess, and Al^3+^ or Cr^3+^ in 400-fold excess or anions (7000-fold excess of Cl^−^, F^−^, NO_3_^−^, 6000-fold of CO_3_^2−^, SO_4_^2−^ and 5000-fold of PO_4_^3−^). This can be attributed to the low uptake capacity of SG-APTMS-N,N-EPANTf_2_ phase towards these species. Based on the above results, one can easily conclude that the newly prepared SG-APTMS-N,N-EPANTf_2_ phase has high selectivity for the adsorption of Zr(IV), and the proposed method can be applied to the detection of Zr(IV) in real samples.

#### 3.5.2. Real Sample Analysis

For checking the reliability of the method, real water samples, including drinking water, tap water, seawater and lake water, collected from Jeddah in Saudi Arabia were subjected to the determination of Zr(IV). The standard addition method was utilized for the analysis of water samples under the same batch conditions. The extraction of Zr(IV) in spiked water samples is reported in Table 3, which results reveal that the extraction of Zr(IV) is in the range of 94.47%–98.96%, confirming the feasibility and suitability of the proposed methodology.

## 4. Conclusions

In this study, a newly developed hydrophobic ionic liquid (N,N-EPANTf_2_) was immobilized on the SG chemically bound amine (SG-APTMS) as a support material. The procedure offered simplicity, and effectiveness and this new reagent provided a selective extraction technique for the determination and extraction of Zr(IV) in aqueous samples with acceptable precision. Performance of the adsorption process of Zr(IV) onto SG-APTMS-N,N-EPANTf_2_ phase followed a Langmuir isotherm model, confirming the growth of a single/monolayer on a homogeneous adsorbent phase. The kinetic isotherm results of Zr(IV) obeyed a pseudo-second order kinetic model. The determination or separation of Zr(IV) by SG-APTMS-N,N-EPANTf_2_ was also not significantly affected by the phase or interferents. The anticipated technique can be employed for the determination of Zr(IV) ions in real water samples with reasonable and reliable results.

## Figures and Tables

**Table 1:** Study of selectivity of 20.0 mg SG-APTMS-N,N-EPANTf_2_ towards various metallic cations.

Metal Ion	Concentration (mg·L^−1^)	*q_e_* (mg·g^−1^)	*K_d_* (mL·g^−1^)
Zr(IV)	2.00	2.49	289.45 × 10^3^
Se(IV)	2.00	0.31	176.94
Cr(III)	2.00	0.33	186.86
Au(III)	2.00	0.30	168.84
Pb(II)	2.00	0.17	92.12
Hg(II)	2.00	0.16	86.90
Cu(II)	2.00	0.12	64.41
Co(II)	2.00	0.01	6.28

**Table 2:** Interferences effect on the extraction of 1.0 mg·L^−1^ Zr(IV) on 20.0 mg SG-APTMS-N,N-EPANTf_2_ (*N* = 3).

Coexisting Ions	Concentration (mg·L^−1^)	Extraction of Zr(IV) (%)
Na^+^, K^+^, NH_4_^+^	3000	98.37
Ca^2+^, Mg^2+^	3000	97.04
Cd^2+^	500	97.58
Cu^2+^	500	96.17
Pb^2+^	500	96.90
Zn^2+^	600	95.24
Fe^3+^	300	93.88
Al^3+^	400	95.33
Cr^3+^	400	94.45
Cl^−^, F^−^, NO_3_^−^	7000	98.57
CO_3_^2−^, SO_4_^2−^	6000	97.39
PO_4_^3−^	5000	98.84

**Table 3:** Determination of Zr(IV) at various concentrations (1.0, 5.0 and 25.0 mg·L^−1^) in real water samples using 20.0 mg SG-APTMS-N,N-EPANTf_2_ (*N* = 3).

Samples	Added (mg·L^−1^)	Unadsorbed (mg·L^−1^)	Extraction (%)
Tap water	1	0.01 (±0.031)	98.79
	5	0.14 (±0.166)	97.27
	25	0.69 (±0.317)	97.26
Lake water	1	0.01 (±0.024)	98.53
	5	0.17 (±0.133)	96.67
	25	0.91 (±0.253)	96.35
Seawater	1	0.02 (±0.079)	97.86
	5	0.23 (±0.182)	95.49
	25	1.38 (±0.421)	94.47
Drinking water	1	0.01 (±0.015)	98.96
	5	0.11 (±0.109)	97.76
	25	0.47 (±0.157)	98.12
